# On the Use of Opium, Bromide of Potassium, and Cannabis Indicum in Insanity

**Published:** 1871-08

**Authors:** T. S. Clouston

**Affiliations:** Medical Superintendent of the Cumberland and Westmoreland Asylum, Carlysle


### On the Use of Opium, Bromide of Potassium, and Cannabis Indicum in Insanity,

ESPECIALLY IN REGARD TO THE EFFECTS OF THE TWO LATTER GIVEN TOGETHER.

By Dr. T. S. Clouston, Medical Superintendent of the Cumberland and Westmoreland Asylum, Carlysle.

[Dr. Clouston states that he has given bromide of potassium alone, or in conjunction with Indian hemp, in fifty-one cases of various kinds of insanity. In all of these cases the drugs had a fair trial, for a sufficient length of time. Very good results were obtained in a considerable number of cases.]

If to a patient whom one has known to have regular attacks of periodic mania for years, we give a medicine at the commencement of an attack, and the patient's excitement ceases, contrary to anything known in the history of the case before, then I think we may fairly conclude that the medicine and the absence of mania are cause and effect. If in a case of mild melancholia at the change of life in a woman, the disorder has existed for a year and a half, if most of the remedies ever before recommended for that class of cases had been tried and failed to do good, and if at last the bromide of potassium procures sound sleep, and immediate visible improvement in appetite, weight, and mental state, surely some credit may be given to it. But if in this same woman its use is intermitted, and all the symptoms at once return, and again immediate improvement follows its employment, so that the patient becomes able to employ herself as she never did before since her illness, and through healthy employment gains in flesh and strength, and gets quite as well in three months as ever she was in her life, surely we cannot deny to therapeutics a cure in the best sense of the term.

Or if a cure cannot be expected, as in a case of general paralysis, if a mixture of bromide of potassium and Indian hemp so subdues intense excitement, that when not taking this medicine the patient is noisy, violent, destructive, sleepless, and rapidly losing weight, and when taking it he is quiet, semi-rational, dresses and eats properly, and remains in this state six weeks, till the disease in its natural course passes into its quiet stage, I think here we have a palliative of great value and importance. Or if an old lady gets irrational, restless, sleepless, and unmanageable by her relatives, and if apparently the last alternative to sending her to an asylum has been tried and failed, until half-drachm doses of bromide of potassium and tincture of Indian hemp is found to subdue and quiet this irritability and restlessness, so that she can be kept quite well at home, for the month or two during which the excitement lasts, and until the ordinary dotage of old age to which this excitement was a prelude, comes on, surely the physician's power was augmented, and the patient was unquestionably the better for the remedy he employed.

In acute mania I seldom found the bromide given alone to do any good, or, indeed, have any perceptible effect. I gave it in all doses up to 120 grains three times a day, and I continued its use in some cases for a few days. But when combined with tincture of cannabis Indica the effects of the mixture were in many cases very remarkable. Sometimes if the excitement was very intense I began with drachm doses of each three times a day, or, in some cases, every three hours for the first day. In the cases in which the effects were good, they usually appeared by the end of the first day of its use. The patients become less restless, the shouting and violence were abated, and at night they slept. The skin, too, which is so often dry in acutely excited patients became more moist, and they perspired freely. The pulse usually lost in force. Indeed, this is the only objection I have to this mixture, that the force of the heart's action is undoubtedly lessened in most cases by it. But I have never seen a single case of syncope, except in one woman who fainted two hours after a dose, but soon recovered. The lessened force of the heart was shown, too, by the paleness of the face and skin generally. After the medicine has calmed the excitement the patient remains confused in mind. The intelligence and coherence of ideas, of course, do not usually return for some time. It is often sufficient if one or two doses per diem are given after the first day or two, and I have stopped its use altogether at that time—the patient remaining free from acute excitement. The greatest advantage of this sedative over every other that I have tried in acute mania was, that these patients took their food as well or better during its use as without it. Every one who has acute mania to treat knows that there are three great risks. The patient's appetite may fail, the excitement may cause complete exhaustion or death, or it may last so long that the power of the brain to become the medium of normal mental manifestations seems to be lost or impaired, and dementia results. There can be no doubt that the patients being got to take a large amount of nourishing food and stimulants is of the very first importance in all cases of acute mania, and it is the great risk of taking away the patients appetite that prevents opium or henbane being more extensively used. Especially is the risk great if we give large doses of opium. It seems to me that the bromide and Indian hemp combined approached more nearly by far than any other drug to our great desideratum in treating acute excitement of the brain, viz., a medicine that will so alter or modify the morbid functions of the brain, that the patient will cease to exhaust all his bodily energy in muscular movement and constant wakefulness, and will at the same time allow the reparative effects of rest and food to act quickly in restoring the normal nutrition of the cerebrum. In some cases complete recovery of the mental powers took place very soon indeed after the excitement was subdued; in others, the confused and incoherent state remained for a long time.

[Dr. Clouston concludes with the following summary :—]

A mixture of one drachm of bromide of potassium with one drachm of the tincture of cannabis Indica is more powerful to allay excitement than any of the other drugs or stimulants tried. It is more uniform and certain in its effects, more lasting, interferes less with the appetite; and to produce the same effect the dose does not require to be increased after long continued use.

Single doses of opium tended to raise the temperature and to lower the pulse; single doses of the mixture above-mentioned to lower the temperature and quicken and weaken the pulse, of bromide of potassium alone to raise the temperature and lower the pulse, of cannabis Indica alone to raise the temperature and quicken the pulse, of whiskey to lower the temperature very much and slightly to quicken the pulse, and of beef tea to lower the temperature in the least degree and to lower and strengthen the pulse.

By giving bromide of potassium and cannabis Indica together, not only is the effect of either given separately immensely increased, but the combination has an essentially different action from either of them given alone.

Bromide of potassium alone can subdue the most violent maniacal excitement, but only when given in immense and dangerous quantities, and its effects are so cumulative while so given, that after they have once begun to appear they increase for days after the medicine has been stopped, almost paralysing the cerebrum and sympathetic.

To produce sleep in mild excitement, one drachm of the bromide of potassium is about equal to half a drachm of laudanum. To allay maniacal excitement, forty-five grains of the bromide and forty-five minims of the tincture cannabis are rather more than equivalent to a drachm of laudanum.

Seven cases of chronic mania were treated for twelve weeks with opium, in doses rising gradually from twenty-five minims of the tincture up to ninety minims three times a day, and the results noted. After getting no medicine for several months the same cases were treated with a mixture of bromide of potassium and cannabis Indica in gradually increasing doses, and the results noted and compared with those of the opium treatment.

Under the opium treatment the patients all lost in weight continuously; their morning temperature was lowered and also their evening temperature, but the latter (which was too high, and its being high was a bad sign) very slightly, and their pulse was decreased in frequency. The opium allayed the excitement in the larger doses, but it soon lost its effect.

Under the bromide of potassium and cannabis Indica treatment the patients only lost in weight very slightly for the first six weeks, and after that they gained, their weight being more at the end of eight months' treatmant than it was to begin with. Their appetites were not interfered with. Their temperature fell, especially their evening temperature, and the pulse was slightly increased in frequency and weakened in force, while the excitement was subdued, and the medicine showed no signs of losing its effect, even after being thus used for eight months. The maximum of good effects and the minimum of the ill effects of a sedative drug were thus obtained by using the bromide of potassium and the cannabis Indica in combination.

The bromide of potassium alone may be continued for months in doses of half a drachm three times a day, and the patients gain in weight and remain healthy in body.

Cannabis Indica being a diuretic, and the bromide of potassium being carried off by the kidneys, it is probable that the former in that way helps to prevent the cumulative action of the latter when given alone.

When the two are given together, the first symptoms developed are those of the cannabis Indica, but these soon merge into a state of drowsy calmness of the nervous system which is in all respects the opposite of nervous irritability.

Fifty-one cases of various forms of insanity were treated by bromide of potassium alone or along with Indian hemp, and the results were that eighty per cent of these were benefitted more or less in some way, and twenty-five per cent. were most decidedly benefitted.

The milder cases of puerperal and climacteric insanity were sometimes remarkably benefitted by drachm doses of the bromide of potassium given at night.

In some of the cases of acute mania the excitement was subdued in a few days by the bromide combined with Indian hemp in doses of from half a drachm to a drachm of each given three times a day.

In some cases of periodic mania and general paralysis all the worst symptoms of maniacal excitement were allayed by giving a mixture of bromide of potassium and cannabis Indica in doses of from half a drachm to a drachm and a half of each three times a day. This was continued in one case for nine months with the best effect.

In three cases of periodic mania, attacks were cut short by a mixture of the two medicines, or by the bromide alone. In one of these complete recovery followed.

Fewer cases of simple melancholia were benefitted by the bromide alone or along with Indian hemp than any other form of insanity. Some were made worse by them, but in one case of this disease where there was great excitement and hallucination of hearing and suspected organic disease of the brain, the combination gave immediate and complete relief of all the symptoms for four months.

One case of senile mania was successfully treated at home by a mixture of the bromide of potassium and tincture of cannabis Indica, when she was to have been sent to an asylum. It seems probable that some such cases, and also patients with short attacks of mania might be treated by the same medicines at home, when at present they have to be sent to lunatic asylums, on account of the want of such a safe and powerful sedative.—*Brit. and For. Med.-Chir. Review.—Braithwaite's Retrospect.*

